# A Triple Perspective on the Triple Visit: Protocol for a Mixed Methods Evaluation of the Implementation of a Pediatric Multidisciplinary Clinic Model for Inflammatory Bowel Disease

**DOI:** 10.2196/72670

**Published:** 2025-09-12

**Authors:** Aisling Curtin Wach, Yasmin Lalani, Alexandra Christofides, Pete Wegier, Lara Hart

**Affiliations:** 1 Humber River Health Research Institute Toronto, ON Canada; 2 Social & Behavioural Health Sciences Division Dalla Lana School of Public Health University of Toronto Toronto, ON Canada; 3 Humber River Health Toronto, ON Canada; 4 Department of Family and Community Medicine University of Toronto Toronto, ON Canada; 5 Institute of Health Policy, Management, and Evaluation Dalla Lana School of Public Health University of Toronto Toronto, ON Canada; 6 Department of Paediatrics Division of Paediatric Gastroenterology McMaster University Hamilton Canada; 7 Department of Paediatrics University of Toronto Toronto Canada

**Keywords:** child-centered methods, Crohn disease, health maintenance, mental health, multidisciplinary care model, pediatric inflammatory bowel disease, patient perspectives, parent perspectives, rapid access clinic, ulcerative colitis

## Abstract

**Background:**

Given the impact of inflammatory bowel disease (IBD) on both physical health and psychosocial functioning, the management of pediatric IBD requires a multidisciplinary approach that provides holistic care, rapid access, and health education to young patients with IBD. Yet despite recommendations, a formalized clinic model for holistic pediatric IBD care remains an unmet need for this population.

**Objective:**

The objective of this study is to comprehensively evaluate the implementation of a new clinic model aimed at providing holistic care, rapid access, and health education for pediatric patients with IBD.

**Methods:**

This study uses a multiperspective mixed methods design, incorporating both qualitative and quantitative data collected simultaneously from clinicians, parents or guardians, and pediatric patients. The quantitative component includes a pre-post implementation chart review, structured observations of clinical practices, and surveys tailored to pediatric patients and their parents or guardians. The qualitative aspect encompasses semistructured interviews with clinicians and parents or guardians as well as play-based interviews with patients. Triangulation will be used to converge these different methodologies and to produce a holistic evaluation of the IBD clinic.

**Results:**

This study was approved by the Humber River Health Research Ethics Board on December 23, 2024. Recruitment for this study began on January 30, 2025, and we anticipate a data collection end date of December 2025.

**Conclusions:**

Given that this clinic model is relatively new, this protocol will provide sound methodological direction for other studies that aim to evaluate or implement this type of model in their setting. The evaluation itself will provide comprehensive insights into both the successes and challenges of implementing a multidisciplinary approach that provides a holistic model of care for pediatric patients with IBD. As such, this evaluation will be foundational in providing an applied understanding of the impact and effectiveness of this holistic approach in pediatric patients with IBD.

**International Registered Report Identifier (IRRID):**

PRR1-10.2196/72670

## Introduction

### Background

Inflammatory bowel disease (IBD) is a chronic and relapsing inflammatory condition that affects not only the patient’s physical health but also their quality of life and social functioning [1,2]. Indeed, patients with IBD reportedly experience increased anxiety and mental health concerns compared with the healthy population [3], in addition to a disease course that is often unpredictable and marked by a waxing and waning pattern, despite surgical intervention and optimal medication [4]. In children, IBD can also affect growth and pubertal development [5]. As such, they are a challenging patient population to manage, which poses a significant burden on health care costs and resources. In Canada, specifically, pediatric IBD has been increasing steadily over the past 20 years [6], and it is expected to exponentially increase in the next 10-20 years [7].

To facilitate the management of complex patients with IBD, recommendations have been put forth to standardize and improve access to IBD subspeciality care. For example, Crohn’s and Colitis Canada recently supported the “Promoting Access and Care through Centres of Excellence” (PACE) program. PACE included a group of IBD experts, nurses, and patients, who sought to develop a portfolio of quality indicators important for the establishment of an IBD clinic [8]. By using the PACE principles to develop an IBD center of excellence, clinicians from McGill University reported that only 6.6% of patients were steroid dependent and 48.8% of patients were on biologic medications following the IBD center’s establishment [9,10]. In addition, the implementation of their IBD rapid access clinic improved health care delivery, replaced many invasive investigations (eg, colonoscopy and endoscopy) with less invasive monitoring methods (eg, fecal calprotectin and c-reactive protein test), and reduced emergency department visits and associated costs [11]. Notably, patients with IBD who presented to the hospital first (n=135) during an acute crisis costed health care an average of CAD $1885 (US $1360) per visit, excluding admission costs, and use of cross-sectional imaging occurred in 65.7% of these visits. This contrasts markedly with the CAD $403 (US $290.93) average cost for patients who presented to the IBD rapid access clinic first (n=488) and the use of cross-sectional imaging was 6%. Despite the guidance provided by PACE and positive impact of the rapid access IBD clinic at McGill, community IBD clinics with rapid access components, including for pediatric IBD, are still uncommon in Canada.

Beyond providing rapid access to patients with IBD, it is also important to provide health education, health maintenance checks and mental health assessments to children with IBD [12]. Education is typically focused on the disease itself, its management, and how to cope with chronic illness [11]. Health maintenance emphasizes ensuring that children with IBD have (1) appropriate growth which is monitored by an IBD nurse and dietician; (2) good nutritional status that includes improving or normalizing vitamin D levels, iron status, and other micronutrients; (3) excellent bone health that includes checking bone age and performing dual-energy x-ray absorptiometry (measures bone density) if they have been exposed to steroids; and (4) up-to-date vaccines [13]. Mental health status should also be assessed on a regular basis, both at baseline and at routine intervals, to ensure early referrals to appropriate providers and prevent poor outcomes associated with anxiety and depression [14]. By providing such services, a pediatric IBD clinic ensures a holistic approach to the management of IBD, while simultaneously preparing children for managing their disease in the future. Subsequently, this improves patients’ management of disease and their self-efficacy and thus provides the building blocks and foundation for a successful transition to adult care [11,15].

To address the significant unmet need of a holistic clinic model of care that provides rapid access to pediatric patients at Humber River Health (HRH), the Outpatient Paediatric IBD Clinic at HRH aimed to achieve several aims within their clinic: (1) Establish a community IBD center with a rapid access IBD clinic based on the guidance provided by the PACE quality indicators. (2) Establish a multidisciplinary community clinic. This provides patients with a biannual visit, referred to as the Triple Visit, where patients would be reviewed by the gastroenterologist (GI), nurse practitioner, and dietician during their visit to the clinic. (3) Establish a mental health–screening program for pediatric patients within the clinic to allow for the proactive identification of patients who need additional supports and resources such as social work, psychology, adolescent medicine, and so on. (4) Develop a health maintenance checklist with proactive management strategies such as bone health, growth assessment, vaccine status, and so on.

### Aims of the Study

To understand the impact of the newly implemented Triple Visit and clinic model of care on HRH’s Outpatient Paediatric IBD Clinic, we aim to comprehensively evaluate its implementation from the triple perspective of patients, parents or guardians, and clinicians. We will evaluate the clinic’s efficacy in (1) improving access to new or unwell patients, (2) improving health maintenance for children with IBD, (3) effectively identifying mental health concerns in this specific population, and (4) satisfying patients’ needs.

Our specific research questions are as follows: (1) What is the impact of the new IBD clinic model of care on patient outcomes such as the use of steroid and biologic medications, health maintenance, and mental health? (2) What is the impact of the new IBD clinic model of care on improving patient access to care and health care usage in general? (3) Are patients and their parents or guardians satisfied with this new model of care? (4) What are the barriers and facilitators experienced by different clinician roles in implementing the new clinic model?

### Study Design and Procedures

**Figure 1:**
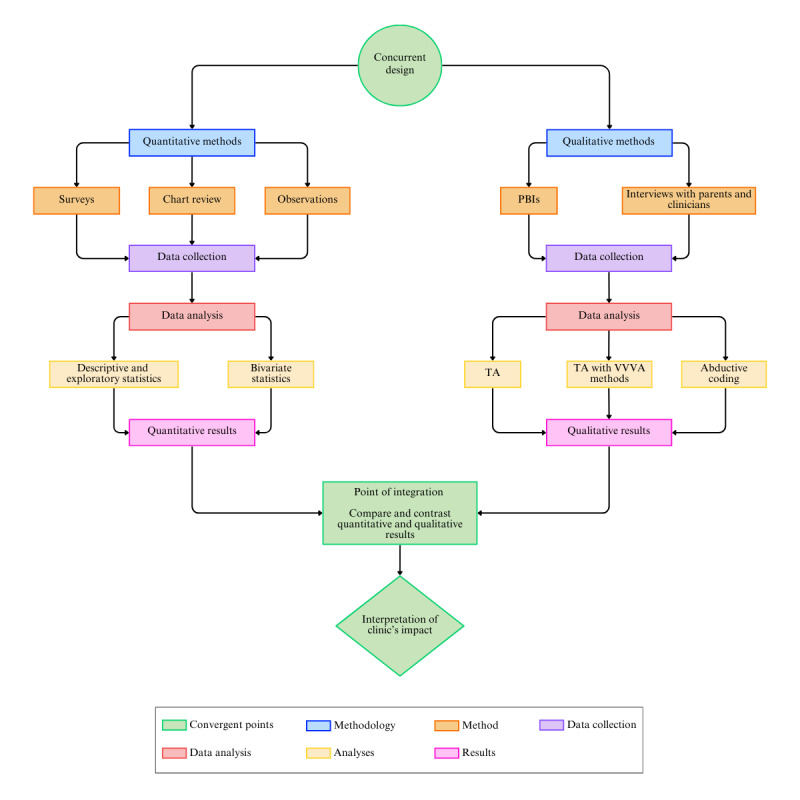
Flowchart illustrating the steps in the convergence model of triangulation design deployed for this evaluation. PBIs: play-based interviews; TA: thematic analysis; VVV A: visual verbal video analysis.

This study is a multiperspective concurrent mixed methods design, incorporating both quantitative and qualitative data collected simultaneously from clinicians, parents or guardians, and pediatric patients [16]. Data will be triangulated to integrate these different methodologies to produce a holistic evaluation of the IBD clinic and its newly implemented components (Figure 1). The quantitative phase will involve a pre-post implementation chart review, structured observations of clinical practices, and surveys tailored to pediatric patients and parents or guardians. The qualitative aspect will encompass semistructured interviews with clinicians and parents or guardians, respectively, as well as play-based interviews (PBIs) with patients. Qualitative data collection and analysis will be guided by the COREQ (Consolidated Criteria for Reporting Qualitative research) standards [17].

By triangulating both qualitative and quantitative methods with an equal weight on each [18,19], we aim to offer a nuanced portrayal of the IBD clinic’s efficacy in achieving its aforementioned aims, in addition to illuminating any of the barriers and facilitators influencing the implementation of the new clinic model. The integration of diverse data sources through triangulation will not only enhance the validity of our findings but also provide a more comprehensive understanding of the clinic’s dynamics, ensuring a well-informed basis for future [20] enhancements and improvements to the IBD clinic.

## Methods

### Participants and Recruitment

To comprehensively evaluate the IBD clinic’s new model of care, including the Triple Visit*,* participants will be recruited from three different perspectives: (1) patients, (2) parents or guardians, and (3) clinicians, including GI physicians, nurse practitioners, dietitians, and mental health professionals such as social workers and psychologists. We will deploy a parallel sampling design [21] in that the samples from the quantitative and qualitative components may be different but are drawn from the same database within the IBD clinic. This sampling design was chosen to increase the uptake in recruitment in a small clinic.

### Pediatric Patients and Parent or Guardians

During IBD clinic outpatient appointments, all parents or guardians accompanying pediatric patients in attendance at HRH’s Outpatient Paediatric IBD Clinic will be asked by the clinic’s GI physicians and nurse practitioner whether they consent to being contacted by the hospital’s research institute in relation to an evaluation of the clinic’s Triple Visit model of care. Study brochures outlining research activities will be provided to parents or guardians during the appointment, in addition to study information cards tailored to our pediatric population. Subsequently, a research team member will connect with parents or guardians who consent to being contacted via their preferred means of communication (ie, email or phone) and will be invited to take part in research activities evaluating the clinic. Patients and parents or guardians can choose to participate in all, some, or none of the research activities. Moreover, patients and parents or guardians will not be recruited as dyads, and therefore one does not necessitate the other for participation. Participants who participate in and complete research activities will be compensated with an e-gift card of their choice from a list of available gift cards and the value of the compensation will correspond to the activity completed. Patient and parent or parent survey compensation value will be CAD $10 (US $7.22), patient and parent or guardian interview compensation value will be CAD $15 (US $10.83), and clinician interviews will be CAD $5 (US $3.61). Patients and parents or guardians who complete a survey and an interview will receive a CAD $10 (US $7.22) bonus for completing both.

For patients, the inclusion criteria will consist of being aged 8-18 years, a patient of the HRH’s Outpatient Paediatric IBD Clinic between January 2021 and December 2024, free from cognitive impairment, verbally fluent, and proficient in English. Patients who do not provide assent or whose parents or guardians do not consent to their participation will be excluded. For parents or guardians, the inclusion criteria will consist of being the parent or guardian of a current pediatric patient of HRH’s Outpatient Paediatric IBD Clinic, free from cognitive impairment, and proficiency in English. For the quantitative phase, we aim to recruit the maximum number of patients (ie, ~80) and their parents or guardians (ie, ~80). For the qualitative phase, we aim to recruit 15 patients, in addition to 15 parents or guardians. For interview studies, a sample size of 15 has been considered to be a sound starting point with the understanding that the final sample size may be adjusted as the study progresses [22].

### Clinicians

For the semistructured interviews, we will invite all clinicians who provide direct care to HRH’s Outpatient Paediatric IBD Clinic patients to participate. This will include GI physicians, nurse practitioners, dieticians, and mental health professionals such as social workers and psychologists. We anticipate that the sample size will be approximately 6-8 clinicians. A combination of convenience and purposive sampling will be used [23], so that all clinician roles in the IBD clinic are represented. This approach will capture the full range of clinician perspectives ensuring that depth of understanding is foregrounded without the need for further recruitment. The director of the Maternal Child Health department will send an email to clinicians who provide IBD care to inform them of the study and the invitation to participate in the interview. To mitigate any coercion, the email will include the contact details of the qualitative methodologist (QM) so that interested clinicians can contact them themselves to learn more about the study and potentially self-enroll.

For the structured observations of clinical practices, we will not be recruiting staff or patients per se but rather aiming to observe appointment types such as first visit, last visit or transitioning to adult care, and Triple Visit. A memo will be sent from the clinic lead to clinic staff, notifying them that observations will be conducted throughout the study duration. Research staff will work with clinicians to pick appropriate times to observe clinical activities based on appointment type and with the consent of parents or guardians.

### Measures and Qualitative Methods

No fixed order will be prescribed for participation in patient- or parent or guardian–facing research activities as this will allow patients and parents or guardians the flexibility to change their mind about what research activities they wish to be a part of. As such, some patients may start with the patient survey, while others may start with the PBI. Similarly, some parents or guardians may begin with the parent or guardian survey, while others may begin with the semistructured interviews. Direct observations, chart reviews, and clinician interviews will flexibly occur over the course of the study. A battery of quantitative measures delivered via patient and parent or guardian surveys, as well as chart reviews and structured observations, will be deployed to quantify and measure the impact of the new clinic model on patient outcomes, in addition to satisfaction with the model of care. Qualitative methods will be used to collect data from clinicians, parents or guardian, and pediatric patients regarding their experiences and perceptions of the new clinic model, including the Triple Visit, as well as their recommendations for improvement. Semistructured interviews will be conducted with clinicians and parents or guardians and a PBI framework method will be used with patients. See Table 1 for a summary of the methods and data sources that will be used.

**Table 1:** Summary of methods, data sources, and purpose.

Methodology and method		Data source		Data examples		Purpose
**Quantitative**						
	Survey	Parents (and on behalf of patients)		Demographics		Contextualize findings
	Survey	Patients		Transition readiness, self-efficacy, IBD^a^ knowledge, service satisfaction, quality of life, and mental health		Understand patient well-being, experiences, and satisfaction in relation to the new clinic
	Survey	Parents		Quality of care, IBD knowledge, transition readiness, health-related quality of life, and parenting stress		Understand parent well-being, experiences, and satisfaction in relation to the new clinic
	Chart review	Patient EMRs^b^		Tests or procedures ordered, prescribed medications, dosage, and number of contacts		Understand changes in clinical outcomes pre-post implementation
	Structured observations of PACE^c^ quality indicators	Clinical encounters, patient EMRs, and consult with clinicians survey		PACE quality indicators [8]		Assess how well the clinic aligns with established guidelines linked to positive patient outcomes
**Qualitative**						
	Semistructured interviews	Parents		Understanding of the Triple Visit, access to the clinic, mental health, health maintenance, and satisfaction with clinic		Garner an in-depth account of parent experiences and satisfaction with the clinic
	CFIR^d^ interviews	Clinicians		Rationale for clinic, advantages of clinic, adaptability of clinic, receptivity to clinic, and self-efficacy		Garner clinician barriers and facilitators with implementing new model
	Play-based interviews	Patients	Feelings toward the clinic, how helpful are the clinicians, and what would be even more helpful		Garner an in-depth account of patient experiences and satisfaction with the clinic	

^a^IBD: inflammatory bowel disease.

^b^EMRs: electronic medical records.

^c^PACE: Promoting Access and Care through Centres of Excellence.

^d^CFIR: Consolidated Framework for Implementation Research.

### Demographics Survey

Patient- and disease-related demographics, as well as parent or guardian demographics, will be collected once, at the time of first participation. Parents or guardians will be responsible for completing demographic information on behalf of themselves and their child via a link sent to them for a demographics survey in Qualtrics. Demographic questions include but are not limited to age, sex, and gender for both the patient and the parent or guardian, as well as the first 3 characters of their postal code, so that researchers can map how far patients travel to receive this specialty care.

### Chart Review of Clinical Outcomes

To understand the impact of the new clinic model of care, including the Triple Visit, on patient outcomes and health care usage (eg, number of contacts with nurse practitioner and tests and procedures ordered), as well as ascertaining whether new clinic components that follow PACE guidelines are being consistently implemented, health maintenance processes are followed, and identified concerns addressed, a pre-post implementation chart review will be undertaken. As the IBD clinic’s new model of care began January 2023, the preimplementation chart review will capture patients who attended the clinic in January 2021 to December 2022, which will then be compared with the postimplementation chart review of patients who attended the clinic from January 2023 to December 2024. Electronic medical records in Meditech will be accessed to complete this review. Variables of interest include prescribed steroid and biologic medications (including dosage), height and weight, and ordered tests. Three data extractors will complete the chart review. Extractor 1 will review 50% of the charts and extractor 2 will review the other 50%. Extractor 3 will re-extract 25% of the charts from each of the other reviewers and interrater reliability will be calculated using the intraclass correlation coefficient.

### Pediatric Patient and Parent or Guardian Surveys

#### Pediatric Patient Survey

To reduce the time and logistical burden of visiting HRH outside of their IBD clinic appointments, participants will be invited to take part in a web-based survey to assess their satisfaction with the new model of care in the IBD clinic, as well as the impact the clinic has had on patients’ knowledge of IBD, transition readiness, self-efficacy, mental health, and health-related quality of life (HRQOL). The web-based survey will be conducted in real time using Zoom (Zoom Communications, Inc), and participant responses will be captured in Qualtrics. Older teenagers may be given the option to complete the web-based survey unassisted via a link sent to their parent or guardian’s email address. PowerPoint slides will be used to display and support the interactive delivery of survey measures. The PowerPoint slides will be appropriately tailored to the age range, as necessary, and measures within the survey will be displayed in an order that places the more difficult questions in the middle, such as questions pertaining to mental health. This is to help children to finish the survey feeling positive. As we project that the survey will take 20-30 minutes for participants to complete, there is the potential for participants to experience survey fatigue. To mitigate against such effects, participants will be offered breaks at intervals throughout the survey, and they will have the opportunity to watch a short age–appropriate cartoon or TV show or to do something of their own choosing such as grabbing a snack. Children who complete their survey on the web via Qualtrics without researcher support will be prompted within the survey to take a break at the same intervals.

##### Transition Readiness

Patients’ readiness to transition from pediatric to adult care will be assessed using the Self-Management and Transition to Adulthood with Rx = Treatment (STARx) Questionnaire [24]. The STARx Questionnaire is an 18-item questionnaire, targeting the following 6 subdomains integral to transition readiness: medication management, provider communication, engagement during appointments, disease knowledge, adult health responsibilities, and resource utilization. All questions are presented to patients in Likert scale format. For example, patients will be asked to indicate on a 5-point Likert scale from 1 (never) to 5 (always), “How often do you take your medications on your own?” and on a 5-point Likert scale from 1 (nothing now) to 5 (a lot), “How much do you know about your illness?” For this present investigation, this tool will help identify gaps not only in skills and knowledge in individual patients but also gaps across the clinic population or where they are doing well as a whole.

##### Patient Self-Efficacy

IBD Self-efficacy Scale for Adolescent and Young Adults is a 13-item questionnaire to assess IBD-specific self-efficacy among patients [25,26]. The scale touches on 4 key areas of disease management including managing medical care, managing daily life with IBD, managing emotions, and managing the future with IBD. Patients are asked to rate their agreement with statements such as “I worry about how much IBD will affect my future” and “I know what to do when I think a flare is starting,” using a 5-point Likert scale from 1 (completely disagree) to 5 (completely agree). As self-efficacy is an important predictor of health outcomes in chronic illness, scores from this scale will be compared with the HRQOL measures, among other measures and variables.

##### Patient Knowledge of IBD

Patients’ knowledge of IBD will be assessed via the Inflammatory Bowel Disease—Knowledge Inventory Device 2 (IBD-KID2) [27]. The IBD-KID2 is a 15-item assessment tool investigating patients’ understanding of IBD across various knowledge domains such as general IBD, treatment, lifestyle, and nutrition. The tool consists of 6 multiple-choice questions and 9 true or false questions, with each correct answer receiving a score of 1, for a maximum total score of 15. Patient knowledge of their IBD is integral to their treatment adherence and development of self-management skills. Thus, similar to the transition readiness scale, the IBD-KID2 will help identify gaps in knowledge among patients of this IBD clinic, which could adversely impact their disease trajectory. IBD knowledge scores will also be compared with their transition readiness scores, HRQOL scores, and parents or guardians’ knowledge of IBD scores, among other measures.

##### Patient Service Satisfaction

Pediatric patients’ satisfaction with the IBD clinic and its new model of care will be assessed via the Youth Satisfaction Questionnaire (YSQ) [28]. The YSQ is a tool that was developed to assess children’s general satisfaction with a service, as well as their satisfaction with specific aspects of the service and activities they are involved in.

To measure general service satisfaction respondents are asked to rate their agreement with 5 statements, such as “Did you get the help you wanted?” and “Have the services helped you with your life?,” using the response options (1) “yes,” (2) “somewhat,” and (3) “no.” Satisfaction with specific service components and activities is measured by asking respondents to assign a grade (A through F) to the specific services and activities they were engaged with. For this project, the specific services and activities list has been generated in collaboration with the research team and IBD clinic care team. Children will be asked to assign a grade to 5 service aspects, such as “medication management” and “access to clinic.” Results from the YSQ will be compared with scores of HRQOL and parental or guardian satisfaction, in addition to informing how the clinic is doing against the PACE quality indicators.

##### Patient HRQOL

The IMPACT-III [29] will be used to assess patient’s HRQOL. In addition to indicating the impact of disease burden on health, HRQOL is an important measure of assessing the efficacy of medical care. The IMPACT-III is a 35-item questionnaire that measures patients’ views on their health, including their physical well-being, emotional functioning, social functioning, and body image. Questions include, “How much energy did you have during the week?,” “Are you embarrassed because of your bowel condition?,” and “How much has your stomach been hurting over the past two weeks.” For each question, respondents are provided 5 possible answers ranging from 1 (eg, “Not at all”) to 5 (eg, “Hurting very much”). HRQOL will be used to assess how well patients in the clinic are doing as a whole and compared with their scores on measures related to mental well-being, in addition to other measures and variables such as their parents’ scores on the parent version of the measure described in the “Pediatric Patient Survey” section*.*

##### Mental Health and Well-Being

The Child Attitudes Towards Illness Scale (CATIS) [30] will be used to assess patients’ attitudes toward their illness. Pediatric patients’ perceptions of their illness can be pivotal in influencing adjustment to their condition, particularly when there is a stigma attached, as is the case for those with IBD. Negative feelings about a chronic condition can lead a child toward maladaptive coping behaviors, which in turn can lead to a more negative adaption to their condition compared with those with positive attitudes. The CATIS uses 5-point Likert scales ranging from 1 (very sad/never/very unfair) to 5 (very happy/very often/very fair) and includes questions, such as “How good or bad do you feel it is that you have [insert illness, e.g., ulcerative colitis/Crohn’s disease]” and “How often do you feel sad about being sick?”. Total sores can range from 13 to 65, with higher scores indicating more positive attitudes. Scores from the CATIS will be compared with other patient measures such as HRQOL.

The Generalized Anxiety Disorder screener (GAD-7) [31] will be administered to patients to screen for anxiety and explore whether the IBD clinic has been achieving its aim of proactively identifying patients who require additional mental health supports. The GAD-7 is a 7-item questionnaire that asks respondents to consider how often over the past 2 weeks they have been bothered by a particular problem, such as “Feeling nervous, anxious or on edge” and “Trouble relaxing.” Response options include 0 (not at all), 1 (several days), 2 (more than half the days), and 3 (nearly every day). Scores ≥10 indicate moderate anxiety.

The Patient Health Questionnaire—9 (PHQ-9) [32] will also be administered to patients to screen for depressions and, similar to the GAD-7, explore whether the IBD clinic has been achieving its aim of proactively identifying patients requiring additional mental health input. The PHQ-9 is a 9-item questionnaire that asks respondents to consider how often over the past 2 weeks they have been bothered by a particular problem, such as “Feeling down, depressed, or hopeless” and “Trouble concentrating on things, such as reading the newspapers or watching television.” Similar to the GAD-7, the PHQ-9’s response options include 0 (not at all), 1 (several days), 2 (more than half the days), and 3 (nearly every day). At the end of the questionnaire participants are asked to indicate how difficult these problems have made it for them in their daily life. Response options range from “Not difficult at all” to “Extremely difficult.” Scores ≥10 indicate moderate anxiety.

##### Desirable Responding

As this survey is asking children and young adolescents to respond to questions regarding sensitive topics such as their symptoms and experiences of IBD, as well as their mental health, there is the potential for participants to respond in socially desirable ways. Thus, the Short Children’s Social Desirability Scale [33] has been included to identify and control for biased responses in patient self-reports. The Short Children’s Social Desirability Scale includes 14 questions to which participants can provide a “yes” or “no” response, such as “Have you ever felt like saying unkind things to a person?” and “Do you always listen to your parents?”

#### Parent or Guardian Survey

Similar to the pediatric patient survey, parents or guardians will be invited to take part in a web-based survey to assess their satisfaction with the IBD clinic’s new model of care, the impact the clinic has had on their knowledge of IBD, and the impact the clinic has had on their child’s well-being. Unlike the pediatric patient survey, parents or guardians will be sent a link to the web-based survey in Qualtrics and will be given a 2-week window to complete the survey at a time of their convenience.

##### Parent or Guardian Service Satisfaction

To measure parent or guardian satisfaction with the IBD clinic, the Quality of Care Through the Patient’s Eyes—Inflammatory Bowel Disease [34] questionnaire developed by the Netherlands Institute for Health Services Research is being adapted. The Quality of Care Through the Patient’s Eyes—Inflammatory Bowel Disease is a 23-item questionnaire that contains 10 generic-specific and 13 disease-specific questions assessing the following 8 dimensions of care: accessibility, costs, accommodation, continuity of care, courtesy, information, competence, and autonomy. Participants are asked to rate the importance of each aspect of care using a 4-point scale (1 “no,” 2 “not really,” 3 “on the whole, yes,” and 4 “yes”) and then to rate the performance of the medical setting and workers based on their experience using the same 4-point scale. Subsequently, a Quality Impact Index is derived for each item, which combines the effect of importance and performance. Note that a Quality Impact Index score of <9 is considered suboptimal patient satisfaction as it is widely held that 90% of patient populations are satisfied with quality of care. To fit the parent format, question structure has been modified as necessary to replace items such as “my” with “me and my child” or “my child’s.” For example, the statement, “Doctors, nurses, and other care works should have a good understanding of my problems” will be modified to read, “Doctors, nurses, and other care workers should have a good understanding of my child’s problems.” Similarly, questions have been edited to reflect the IBD clinic; thus, some statements have either been removed or modified. For example, in the original version, participants are asked to respond to questions about their general practitioner and specialist, respectively. In this survey, questions pertaining to general practitioners have been removed and questions regarding the patient’s specialist are reworded to reflect the IBD specialist team. Parents or guardians will also complete the same grading activity their children complete from the YSQ, that is, parents or guardians will assign a grade (A through F) to the specific services and activities their child has engaged with.

##### Parent or Guardian Knowledge of IBD

Parents or guardians’ knowledge of IBD will be assessed via the IBD-KID2 described in *the patient survey.* This measure will allow for comparison between patients and parents or guardians, as well as investigating the relationship between parents and guardians’ knowledge of IBD, service satisfaction, parenting stress, and so on.

##### Parent or Guardian Perception of Child’s HRQOL

The parent IMPACT-III (IMPACT-III-P) [35] will be used in conjunction with the IMPACT-III [29] described in the patient survey to assess patient’s HRQOL. The IMPACT-III-P uses the same 35 items from the IMPACT-III, with personal pronouns modified to fit the parent format. For example, the children’s version asks, “How much energy did you have during the week?” whereas the parents’ version has been modified to ask, “How much energy does your child have during the week?” Parent scores from the IMPACT-III will be compared with patient scores on the same measure.

##### Parent or Guardian Perception of Child’s Transition Readiness

The parent STARx Questionnaire (STARx-P) [36] will be used in conjunction with the child STARx Questionnaire described in the *patient survey* to assess patients’ transition readiness, in addition to comparing potential differences and similarities in patients and parents or guardians’ perceptions of transition readiness. The STARx-P uses the same 18 questions and Likert scale response options as the STARx, with the word “you” being replaced as necessary with “your child” to fit the parent format.

##### Pediatric Parenting Stress

Pediatric parenting stress will be assessed with the Pediatric Inventory for Parents (PIP—Short Form) [37]. Across 13 questions parents are asked to consider over the past 7 days how often and how difficult they experienced and found particular events such as sleeping, attending work, and seeing their child sad. Thus, 13 questions produce 26 responses. A 5-point Likert scale is used for the response options for both “How often” (ie, from 1 “never” to 5 “very often”) and “How difficult” (ie, from 1 “not at all” to 5 “extremely”). Parents’ scores on the Pediatric Inventory for Parents will be compared with their knowledge of IBD and patient HRQOL, among other measures.

### Structured Observations of PACE Quality Indicators

As the implementation of PACE guidelines in other clinics has resulted in positive patient outcomes [9,10], a structured observation of clinical practices in this new model of care at HRH’s Outpatient Paediatric IBD clinic will be conducted. The structured observation will involve direct clinical observation of clinical appointments such as a new patient visit, last or transition to adult care visit, and the Triple Visit, in addition to the review of specific electronic medical record metrics from patients’ charts, measurement of patient satisfaction as per the patient survey, and consultation with clinical staff, as necessary. The clinic’s pediatric GI has been consulted to determine the applicability of each guideline to the pediatric setting. As such, 43 of 45 indicators that cover structure (the inclusion of particular health care professionals in the clinic, etc), process (performing tests and prescribing particular medications based on certain criteria, etc), and outcome quality indicators (patient health care usage, patient satisfaction, etc) have been included for this structured observation. To mitigate against observer bias, particularly for direct observations of clinical appointments, and consistency issues across observations, 2 or more methods (ie, direct observation of clinical appointments, chart review, and consultation with clinical staff) will be used, when possible, to validate whether a particular indicator is observed in the clinic’s practices. Three data extractors will be used to extract chart review data and interrater reliability will be calculated using Cohen k.

### Semistructured Interviews With Parents or Guardians and Clinicians

#### Parent or Guardian Semistructured Interviews

Parents or guardians will participate in 1 virtual or in-person semistructured interview. Questions will focus on the key elements of evaluation that include their perspectives on accessing the clinic, mental health, their views on how their child’s health is being maintained, and more general views on their satisfaction with the new clinic model. To round off the interview, parents or guardians will be asked to provide any recommendations to any aspect of their clinic experience.

#### Clinician Semistructured Consolidated Framework for Implementation Research Interviews

For clinicians, the focus will be on garnering their perspectives of how the new clinic model was implemented, paying specific attention to the experiences of the different clinician specialties that comprise the new model. To do so, we will use the Consolidated Framework for Implementation Research (CFIR), a validated 2-versioned framework comprising a taxonomy of constructs that have been shown to be associated with implementation success [38,39]. A defining feature of the Triple Visit is its commitment to coordinated care and collaboration among a multidisciplinary team; as such, attention will be paid to the Characteristics of Individuals domain of the CFIR. This domain aims to uncover contextual information about how those involved in implementing and executing the Triple Visit model make sense of the new initiative and the interplay among other staff within the purview of the intervention. Other CIFR domains included in the interview guide are Intervention Characteristics to obtain data about participants’ knowledge and perceptions of the new clinic model, Outer Setting that focuses on clinicians’ opinions on the patient or parent population, Inner Setting to gather insights on whether the new clinic model is embraced in the hospital context, and Process to understand clinicians’ views on how the new model was implemented. All interview questions aim to uncover the barriers and facilitators associated with implementing the new clinic model and clinicians’ insights on how to improve the service.

### Pediatric Patients and PBIs

We aim to gather insights from patients with IBD about their experience of the IBD Triple Visit clinic visits. Data collection scripts used with patients will be tailored to their developmental stage, guided by a narrative PBI framework that incorporates play and opportunities for creative expression as a vehicle to elicit their responses from the interview guide [40-42]. Previous research on conducting qualitative data collection with young children and adolescents shows that novel methods such as play, drawing, diary writing, or other forms of creative expression are effective at eliciting responses from children in a supportive setting [43]. These methods reside outside an artificial interview context and instead are grounded in a “child’s perspective”—a perspective that has been methodologically overlooked in studies involving children in hospital settings [44]. Approaching interviews with children in a way that reflects their developmental stage enhances the validity of the data and, in turn, their quality [41,45].

A certified child life specialist (CCLS) will facilitate the PBIs. CCLS are health care professionals who are clinically trained in evidence-based techniques to help children feel emotionally safe and calm in hospital settings when undergoing medical procedures and invasive examinations. Thus, the CCLS is well suited to facilitate the PBIs, as they possess the skill set to understand both children’s developmental stage and socioemotional needs. To ensure rigor in data quality, a QM will also be present to take detailed field notes to compliment the audio and video data from the interviews.

#### PBI Procedure

For the PBIs, children will be split into their developmental ages: 8-12 years, 13-15 years, and 16-18 years, which will determine the age-appropriate interview script to use on the interview guide for the PBI. Sessions will be audio and video recorded in a room within the Maternal and Child clinic; however, parents will have the option on the consent form to not have their child video recorded. The video camera will be attached to a laptop and positioned in a specific area of the clinic room to ensure that it does not interfere with the child’s activities during their interview with the CCLS. In line with child interview recording, our aim is to ensure that the experience remains as unobtrusive as possible [41].

The QM will obtain consent from parents and assent from the patients prior to the PBI; it will be reiterated to the patient that their participation is voluntary and that they may stop the PBI at any time. The CCLS will use the interview guide using the age-appropriate script that corresponds to the age of the patient. The interview guide will touch on the following topics: what the children like about coming to the clinic, what they do not like, their thoughts about their care team, what they find helpful about the clinic, and how they would like their clinic experience to be improved. To elicit responses from the patients, they will be invited to choose 1 or more items from an activity portfolio [41] located in the interview clinic room to play or engage with. While the patient is occupied with their activity of choice from the portfolio, the CCLS will consult the interview guide and elicit their responses; having the child “busy” with an enjoyable activity allows for a more a natural conversation with the child to unfold [40,41]. At the end of the PBI, the patient will be invited to choose a small gift of appreciation from a “Thank You Bin” in the clinic room. After the CCLS and QM lead the child back to their parents or guardians in the waiting area, the child will also be offered a gift card.

### Ethical Considerations

This study received HRH Research Ethics Board approval on December 23, 2024 (REB#24-0005). Prior to commencing research activities, a researcher will obtain verbal consent from parents or guardians for the specific research activity being undertaken. Parents or guardians will be responsible for providing consent for their and their child’s participation. Although parents or guardians will provide consent for their child’s participation, all pediatric patients will also be asked at the time of participation whether they assent to participating in the research activity at hand. Age-appropriate assent scripts will be used to describe the specifics of the research activity, including that their participation is voluntary, they have the right to withdraw at any time without penalty, and that their information will be kept private and confidential. If a patient does not assent, they will be withdrawn immediately, thanked for their time, and reassured that this will not impact the care they receive at HRH. Clinical staff who are interested in participating in research activities will be provided with a consent document to review, followed by a verbal read through with a member of research staff. Ample time will be provided for staff to ask the researcher questions before verbally indicating whether they wish to participate. As employees of HRH and the department in which research activities are taking place, some clinicians may feel a sense of pressure to participate, or perhaps even worry about work-related repercussions should they decline an invitation to engage in research activities. Clinical staff will be made aware that their participation is voluntary and not a condition of employment. The possibility of coercion or undue influence will be minimized, as research staff from the hospital’s research institute, rather than staff from within the IBD clinic, will be responsible for recruiting and consenting clinical staff into the study. Participants will receive nominal gift cards as outlined in the “Methods” section as a recognition of their time and contribution. Their nominal value is in line with ethical standards.

### Data Analysis and Sample Size

In line with a concurrent triangulation design, analysis of qualitative and quantitative data will be analyzed separately. Results from both methods will then be integrated and interpreted where appropriate.

### Quantitative Analysis

#### Chart Review

Baseline demographics, clinical history, and outcomes including health care usage will be extracted from the electronic medical records for patients who had an IBD clinic visit any time since January 2021 to December 2024. A prospective chart review will also be conducted to extract post-Triple Visit patient information for those who had their first Triple Visit in 2023-2024. The primary goal of this chart review will be to compare patient outcomes at 6 months before and after their first Triple Visit to understand how the new model of care has affected patient outcomes. The primary analysis will be a matched *t* test, with a 2-sided *P*<.05 as the critical value, effect size of 0.35, and power of 0.8. This estimates a required sample size of at least 67 patients for meaningful analyses.

#### Patient and Parent or Guardian Survey Data Analyses and Sample Size Calculation

The primary analysis will be a correlation between patient service satisfaction scores from the YSQ and HRQOL from the IMPACT-III, with 2-sided *P*<.05 as the critical value, a medium effect size of 0.35, and a power of 0.8. This results in a required sample size of 61 patients. Secondary analyses will be descriptive statistics and bivariate statistics using *t* tests, chi-squared test, and Pearson or Spearman correlations to identify relationships between other variables captured in the survey. Identical analyses will be conducted for the parent or guardian survey; thus, we will aim to recruit 61 parent or guardians, also. Once data are collected from both surveys, bivariate statistics will be used to compare differences between the patients and parent or guardians for the scales present in both surveys.

#### Structured Observation

Descriptive statistics will be used to capture the percentage of PACE Quality Indicators that have been implemented by the clinic, as well as the clinics compliance with these implemented measures.

### Qualitative Analysis

#### Semistructured Interviews With Parents or Guardians

Parent or guardian interview transcripts will be uploaded to NVivo 14 (Lumivero) and will be analyzed thematically, primarily drawing on Braun and Clarke’s 6 steps for analysis [46,47].

#### Semistructured Interviews With Clinicians

For clinician interviews, all transcripts will be uploaded into NVivo 14 where codes for each CFIR construct will have already been predefined from the CFIR taxonomy [38]; interviews will be coded deductively against the CFIR constructs selected from the interview guide. Interviews will also be coded inductively for relevant data that are not captured by the CFIR framework; the CFIR framework is adaptable in that researchers can add project-specific codes during analysis to capture factors that influence implementation but fall outside the predefined CFIR constructs. This abductive approach produces a contextually grounded analysis [48,49].

#### Pediatric Patients and PBIs

Pediatric patient data will be collected in the form of audio and field note data from the play sessions and with video data if the child’s parent or guardian had provided consent for their child to be video recorded. If the children created artwork, block towers, or drawings, these products will not be analyzed as they are not methods per se but rather the evidence that the child engaged in responses to the interview questions that the child answered during the play session.

For children who were video recorded during their play session, these recordings will be analyzed using the 6 steps outlined in the visual verbal video analysis method; the method aligns with a similar approach to classic thematic analysis [47], with an emphasis on selecting key moments in the video to analyze. The steps involve collecting and reviewing data, transcribing, choosing units of analysis, coding data, organizing, and describing data and reporting the findings [50]. For children whose parents or guardians opted out of video recording, their data will consist of an audio recording of the PBI in addition to field notes taken by the qualitative specialist; field notes will be structured using the visual verbal video analysis matrix framework that includes the following topics: general characteristics of the video, multimodal and visual characteristics, and participants in the video and content including emotions, gestures, and discourses [50,51]. Analytic memos will be written by the CCLS and the QM after each patient interview; writing memos immediately after the data collection session will be critical to enhance validity and facilitate consensus discussions during analysis [52].

### Data Triangulation

We anticipate that the datasets from the qualitative and quantitative components that will intersect at the “point of interface” [53] will be the parent satisfaction survey with the parent interviews and the patient satisfaction survey with the patient interviews. With these datasets, we will be guided by Teddlie and Tashakkori [16]: (1) data reduction, (2) data display, (3) data transformation, (4) data correlation, (5) data consolidation, (6) data comparison, and (7) data integration. More specifically, themes generated from qualitative results will be transformed into quantitative variables for comparison with the survey items. This comparison will be an iterative process wherein both datasets will be in conversation with each other to produce a consolidated profile of parent and patient satisfaction of the clinic.

## Results

This study received REB approval in December 2024. Recruitment commenced in January 2025, and is ongoing with an anticipated data collection end date of December 2025. Thus, results are not yet available.

## Discussion

### Anticipated Findings

We anticipate that this mixed methods evaluation of a holistic pediatric IBD clinic model of care that includes a multidisciplinary team Triple Visit, as well as a rapid access component, will provide a strong rationale for a multidisciplinary approach to care for children with IBD, a population that is increasingly prevalent in Canada [7]. Our results aim to demonstrate how the clinic’s accessibility, processes for health maintenance checks, patient and parent or guardian satisfaction, and the monitoring of patients’ mental health will enable a more comprehensive and holistic approach to patient care and management of IBD.

### Limitations

Limitations of this study can be pinpointed. First, data integration of mixed methods concurrent designs is not without flaws [18]. Integrating and interpreting data sources from qualitative and quantitative results may pose challenges as it is possible that not all data sources will be compatible with each other, especially given the chasm between the 2 paradigms and their respective epistemological assumptions [54]. Consequently, we expect that some findings may exist independently within each paradigm rather than fitting together across both; these discrepancies may require further research to resolve them [55]. However, a benefit of this mixed methods approach is where one method has weaknesses or produces a gap in knowledge, the other method will be a strength by filling in those gaps. For example, from a quantitative perspective it is not possible to gather a true measure of pre-post implementation satisfaction among patients and their parents or guardians as satisfaction was not objectively measured prior to the new clinic model’s implementation. By triangulating results from measures in the patient and parent or guardian surveys with the patient PBI and parent or guardian interviews, a fuller understanding of satisfaction pre-post implementation can be derived. Finally, the process of integrating and interpreting the data will be time-consuming as integration cannot occur until all data have been analyzed; generally speaking, qualitative data analysis is labor intensive and, as such, leads to a longer timeline for completion [56,57].

### Conclusions

This protocol will be instructive for other scholars and clinicians of pediatric IBD. Given that the multidisciplinary clinic model is relatively new, we believe that this protocol will provide sound methodological direction for other studies that aim to evaluate or implement this model in their setting. From an implementation science perspective, qualitative results with clinicians will reveal contextual information about barriers and facilitators of putting the model in place in the clinic [38]; these results will show specific details on what factors led to high or low implementation success—data that may prove useful for other clinics that aim to implement a similar model of care. From the perspective of the pediatric IBD clinic at HRH, we anticipate that this evaluation will lay the foundation for further monitoring and improvement of the clinic model, in addition to evaluating this model from a health economist and resource-specific lens.

## Abbreviations

CATIS: Child Attitudes Towards Illness Scale
CCLS: certified child life specialist
CFIR: Consolidated Framework for Implementation Research
COREQ: Consolidated Criteria for Reporting Qualitative Research
GAD-7: Generalized Anxiety Disorder screener
GI: gastroenterologist
HRH: Humber River Health
HRQOL: health-related quality of life
IBD: inflammatory bowel disease
IBD-KID2: Inflammatory Bowel Disease—Knowledge Inventory Device 2
PACE: Promoting Access and Care through Centres of Excellence
PBI: play-based interview
PHQ-9: Patient Health Questionnaire—9
QM: qualitative methodologist
REB: research ethics board
STARx: Self-Management and Transition to Adulthood with Rx = Treatment
VVVA: visual verbal video analysis
YSQ: Youth Satisfaction Questionnaire
